# Public and Healthcare Provider Receptivity toward the Retention of Dried Blood Spot Cards and Their Usage for Extended Genetic Testing in Hong Kong

**DOI:** 10.3390/ijns9030045

**Published:** 2023-08-11

**Authors:** Kiran Moti Belaramani, Cheuk Wing Fung, Anne Mei Kwun Kwok, Shing Yan Robert Lee, Eric Kin Cheong Yau, Ho Ming Luk, Chloe Miu Mak, Matthew Chun Wing Yeung, Olivia Miu Yung Ngan

**Affiliations:** 1 Metabolic Medicine Unit, Department of Paediatrics and Adolescent Medicine, Hong Kong Children’s Hospital, Hong Kong, China; 2 Department of Paediatrics and Adolescent Medicine, Pamela Youde Nethersole Eastern Hospital, Hong Kong, China; 3 Department of Paediatrics and Adolescent Medicine, Princess Margaret Hospital, Hong Kong, China; 4 Clinical Genetics Service Unit, Hong Kong Children’s Hospital, Hong Kong, China; 5 Newborn Screening Laboratory, Division of Chemical Pathology, Department of Pathology, Hong Kong Children’s Hospital, Hong Kong, China; 6 Medical Ethics and Humanities Unit, School of Clinical Medicine, LKS Faculty of Medicine, University of Hong Kong, Hong Kong, China; 7 Centre for Medical Ethics and Law, Faculty of Law and LKS Faculty of Medicine, University of Hong Kong, Hong Kong, China

**Keywords:** newborn screening, inborn error of metabolism, inherited metabolic diseases, dried blood spots, residual dried blood spots, extended genetic testing, biobank, survey, Hong Kong

## Abstract

Dried blood spot (DBS) cards from newborn screening (NBS) programs represent a wealth of biological data. They can be stored easily for a long time, have the potential to support medical and public health research, and have secondary usages such as quality assurance and forensics, making it the ideal candidate for bio-banking. However, worldwide policies vary with regard to the duration of storage of DBS cards and how it can be used. Recent advances in genomics have also made it possible to perform extended genetic testing on DBS cards in the newborn period to diagnose both actionable and non-actionable childhood and adult diseases. Both storage and secondary uses of DBS cards raise many ethical, clinical, and social questions. The openness of the key stakeholders, namely, parents and healthcare providers (HCPs), to store the DBS cards, and for what duration and purposes, and to extended genetic testing is largely dependent on local cultural–social-specific factors. The study objective is to assess the parents’ and HCPs’ awareness and receptivity toward DBS retention, its secondary usage, and extended genetic testing. A cross-sectional, self-administrated survey was adopted at three hospitals, out of which two were public hospitals with maternity services, between June and December 2022. In total, 452 parents and 107 HCPs completed and returned the survey. Overall, both HCPs and parents were largely knowledgeable about the potential benefits of DBS card storage for a prolonged period and its secondary uses, and they supported extended genetic testing. Knowledge gaps were found in respondents with a lower education level who did not know that a DBS card could be stored for an extended period (*p* < 0.001), could support scientific research (*p* = 0.033), and could aid public health research, and future policy implementation (*p* = 0.030). Main concerns with regard to DBS card storage related to potential privacy breaches and anonymity (Parents 70%, HCPs 60%). More parents, compared to HCPs, believed that storing DBS cards for secondary research does not lead to a reciprocal benefit to the child (*p* < 0.005). Regarding extended genetic testing, both groups were receptive and wanted to know about actionable childhood- and adult-onset diseases. More parents (four-fifths) rather than HCPs (three-fifths) were interested in learning about a variant with unknown significance (*p* < 0.001). Our findings report positive support from both parents and HCPs toward the extended retention of DBS cards for secondary usage and for extended genetic testing. However, more efforts to raise awareness need to be undertaken in addition to addressing the ethical concerns of both parents and HCPs to pave the way forward toward policy-making for DBS bio-banking and extended genetic testing in Hong Kong.

## 1. Introduction

The advances in high-resolution tandem mass spectrometry in the 1990s led to a paradigm shift in expanded newborn screening (NBS) based on dried blood spot (DBS) cards [1]. Currently, NBS using a heel prick test, collecting a few drops of blood from the baby’s heel on filter paper, is widely available in many regions and countries [2]. The residual DBS cards can be stored for long periods, and storing these materials represents a wealth of biological data. It offers an excellent opportunity for secondary analytic uses to improve current screening programs, facilitate laboratories’ quality control and assurance, forensics, and expand biomedical research [3,4,5,6]. Studies have also shown that dried bloodspots can also aid disease treatment via early detection of genetic and infectious diseases. DNA is stable in DBS for prolonged periods, making it the ideal candidate to aid early diagnosis of genetic conditions using expanded genetic screening. Studies have shown that DBS cards can aid the diagnosis of congenital cytomegalovirus and neonatal herpes simplex virus infection. Other potential maternally transmissible conditions that could be diagnosed using DBS cards include HIV, Hepatitis B, and C. In utero exposure to potentially toxic environmental agents such as lead, mercury, and dichlorodiphenyldichloroethylene (DDE) are also measurable on DBS cards and could aid diagnosis and etiologic studies of birth defects and developmental disabilities in relation to environmental agent epidemiology [7,8,9].

Storing DBS cards for secondary usage raises clinical, ethical, and social considerations. The most frequently discussed concern is informed consent. A pre-requisite for informed consent is knowledge. Locally, Mak et al. have shown that parents have a deficient knowledge of DBS cards and NBS programs [10]. Van Teeffelen et al. have shown that 81.8% of parents were unaware of the DBS card screening, storage, and use policies in the Netherlands [11]. On the other hand, Tarini et al. showed that parents in the United States (US) demonstrated high trust and support toward storing DBS cards [12], and they were willing to give out their child’s DBS cards only if explicit consent was obtained through an opt-in approach [13,14,15]. Another significant issue is data privacy breaches. Studies showed fears about misusing information for purposes that deviate from their original intended use, driving discrimination by employment, insurance, or medical treatment, inequitable access to healthcare services, and potential bias against individuals [16,17]. However, some medical geneticists argued that these are negligible concerns, as most research uses involve de-identified or anonymized bio-samples [18]. Some evidence suggests that deliberative consent procedures encourage research participation, helping parents understand the process and purpose of the secondary use of DBS samples for research and empowering their need for control over the use of their samples. Other apprehensions about DBS card storage include unauthorized access to information, unclear research purposes, and the absence of data management and governance [16,19].

The rapid development of next-generation sequencing is leading to an evolution in NBS, using DNA material on the DBS card to discover disease genes and diseases at an early age [20]. This technology can be used as a primary screen, especially for those early-onset actionable disorders that lack a good biochemical marker or follow-up testing after an abnormal primary screen. However, on the downside, extended genetic testing can also provide provisional positive or inconclusive information about an individual’s future health, which can significantly impact their lives and the lives of their family members. Studies have shown that although parents were often optimistic about receiving their child’s health information and felt it would be a parental obligation to ensure a healthy life, their initial affective responses toward an inconclusive test result were often ones of distress, shock, and anxiety, mainly due to their inadequate knowledge of screened-for conditions and genetics [21,22].

Policies worldwide vary regarding how and when to consent for biospecimen storage and its secondary uses because of the sensitive information that can be derived from the DBS card [2]. In Hong Kong, there is a DBS-based opt-in NBS program covering 26 inherited metabolic diseases (IMD) and severe combined immunodeficiency which has been available to all newborns at all eight public hospitals with maternity services since 2020 and 2021, respectively. Parents are given information about the NBS program in both the antenatal and postnatal period through the display of videos and distribution of education pamphlets to help them understand the newborn screening program prior to the attainment of written consent. In the current framework, DBS samples will be archived for six months except for quality assurance purposes, and no research purposes will be served. The optimal policy governing NBS technologies is contextually dependent, and empirical research on this topic among the general public and medical professionals is limitedly reported in the local context. The specific study objective is to describe the knowledge, attitudes, and practices toward the storage and secondary use of DBS, including extended genetic testing among parents and healthcare providers (HCPs) in Hong Kong.

## 2. Materials and Methods

### 2.1. Sampling and Recruitment

A cross-sectional, self-administrated survey was adopted at three hospitals, including two public hospitals with maternity services, between June and December 2022 (The survey questionnaires are included in the Supplementary Materials). The study population comprised parents and HCPs.

Parents were recruited using convenience sampling. They were initially approached while they attended the pediatric service outpatient clinic at three public hospitals. The researcher ensured that parents were fully informed about the study. Basic background information on the concepts of the DBS card and its potential were included in the study information leaflet during recruitment. After obtaining written consent, they received a hard copy or an e-copy of the survey. HCPs, including doctors, nurses, dieticians, laboratory technicians, and those in other fields relating to clinical services, were invited to participate in the study using snowballing sampling. The study was emailed to every member of the department. A survey package, including an informed consent form and a paper questionnaire, was also distributed to the HCPs’ office mailbox. Anyone who was incompetent in Chinese or English, or refused to consent, was excluded from the study.

The completed survey questionnaires were either submitted online, returned by mail using a self-addressed stamped envelope, or collected on-site. A reminder was also sent two weeks after the initial email. No incentives for survey participation were provided.

### 2.2. Survey Measures

The survey consists of two main themes, along with demographic information. The first theme aims to gather views on the awareness of NBS for uncommon disorders and assess knowledge, attitudes, and practices toward the storage of DBS cards. A vignette is provided to ensure survey respondent has an equal understanding the study context (Box 1).

Box 1 **Description of Newborn Screening with Dried Blood Spots Card.**

Shortly after the baby infant is born, a health professional will perform a heel prick to collect a few drops of blood on a special card. The Card will be sent to the laboratory for the screening of some rare but serious conditions which could be picked up and treated early with benefit the affected babies in the long run. The information also benefits the paediatric population and other scientific research.

At present, the card is kept for 6 months after the test has been performed for quality assurance purposes and will then be discarded. Other countries, the card will be kept longer (a few years or twenty years or more) for other research purposes.

The domain of questions includes several key areas:

(1) The source of information, which is evaluated by assessing various sources of knowledge, such as discussions with healthcare professionals, media, leaflets, colleagues, and online forums.
(2) Knowledge about the potential benefits of storing a DBS card is assessed by presenting participants with 11 true or false statements. These statements have been developed based on a thorough review of the NBS literature and position statements. A higher score indicates a better understanding of the advantages of storing a DBS card.
(3) Attitudes toward DBS card storage are assessed by examining concerns relating to privacy breaches, data sharing among institutions, linking research information to medical records, lack of immediate individual benefits, and serving secondary research purposes. Each item is rated on a 5-point Likert scale, ranging from 0 (not concerned at all) to 5 (extremely concerned).
(4) Practices are assessed by evaluating participants’ interest in storing their child’s sample after the NBS on a 1-to-10 Likert scale (1 indicating not supportive, and 10 indicating extremely supportive). Additionally, participants are asked how long they believe the DBS card should be stored.

The second theme of the study focused on exploring the inclination toward opting for extended genetic testing after reading the vignette (Box 2). To achieve this, a set of eight scenarios were presented to parents, inquiring whether they would like to receive the results of the extended genetic testing for their child. Similarly, HCPs were asked if they would disclose this information to parents, and both groups responded with a simple “Yes” or “No”. The study also delved into the factors that influence the interest of parents and HCPs in extended genetic testing, which were measured using a 5-point Likert scale, with 1 indicating “not important at all” and 5 indicating “extremely important”. Finally, the participants were asked to express their level of interest in extended genetic testing, either for their child or as an offering, on a scale of 1 to 10, where 1 represents no interest at all, and 10 represents an extremely high level of interest.

Box 2 **Description of Extended Genetic Testing.**

It is possible to study a person’s entire genome extracted from DBS card. This process is called extended genetic sequencing. It may give you information about a person’s risk of having different diseases both in the present and in the future, including childhood diseases such as epilepsy and adult-onset diseases breast cancer or diabetes.

Imagine that you have a recently born baby. Newborn Screening program now offers you the chance to get extended genetic sequencing done for your baby. You would receive the results and would not have to pay for the testing. You can decide whether or not you want the information to be a part of your child’s medical record.

### 2.3. Data Analysis

Descriptive statistics were applied. Analyses specifically focused on the differences between parents and healthcare professionals. Chi-square and t-test were used to compare these two groups. Statistical significance was considered if the *p*-value was less than 0.05. Data were analyzed using SPSS (Version 28; SPSS, Inc., Chicago, IL, USA).

## 3. Results

### 3.1. Demographics Characteristics

A total of 452 parents and 107 HCPs completed and returned the survey. Table 1 describes their demographic characteristics. Among 452 parents, the majority were female (74.3%), Hong Kong Chinese (86.9%), had no religious affiliation (70.4%), had two or more children (56.2%), and did not work in a healthcare-related industry (90.5%). Around three-fifths attained post-secondary school education (62.0%), which is similar to the educational attainment of the general population reported in the 2022 census [23]. Around 62.4% of respondents earned more than the median monthly household income (HKD 29,100) [24]. Over 93.8% of respondents were not part of a consanguineous marriage. There were 161 (35.6%) parents who were taking care of a child with a disease, of whom 40 (8.8%) were suffering from cardiac disease, 21 (4.7%) from hematological disorders, 16 (3.5%) from respiratory diseases, 15 (3.3%) from neonatal diseases, 14 (3.1%) from endocrine diseases, 9 (2.0%) neurological diseases, and 8 (1.8%) from inherited metabolic diseases.

Among 107 HCPs, the majority were female (71%), Hong Kong Chinese (100%), had no religious affiliation (48.6%), and attained post-secondary school education (98.1%). About one-third were doctors (31.8%) and nurses (33.6%). The remaining were allied health professionals, including dietitians, physiotherapists, occupational therapists, and laboratory technicians (34.6%) involved in healthcare provision to patients with uncommon diseases such as IMD. The average number of years of experience/involvement in NBS practice was 5.49 (SD = 7.35).

### 3.2. Awareness of Newborn Screening for Uncommon Disorders

In terms of sources of information, some parents have obtained NBS-related information from more than one medium. Among 452 parents, 237 (52.4%) have heard about NBS, where they rely on health providers as the most credible source of information (61.2%), followed by hospital brochures (37.1%) and online platforms and media (13.9%). Of those 237 responding parents who have heard about NBS, 139 (58.7%) have taken part in an NBS program for uncommon diseases, 56 parents (23.6%) have not taken part in any screening program, and 42 (17.7%) are uncertain.

Among 107 HCPs, 93 (86.9%) have heard of NBS, where they receive test information from colleagues (84.9%), hospital brochures (36.6%), and academic research papers (19.4%). Of those 93 responding HCPs who have heard about NBS, 47 (50.5%) are involved in NBS, performing various duties including informing parents of test results (34%), drawing blood tests (21.3%), providing consultation (19.1%), conducting laboratory analysis (12.8%), hosting educational sessions (6.4%), and facilitating consent procedure (6.4%).

Overall, most parents who have participated in NBS (97.9%), and all HCPs who are involved in NBS (100%), find NBS for uncommon disorders useful.

### 3.3. Knowledge about the Potential Benefits of Storing Dried Blood Spot Cards

**Figure 1:**
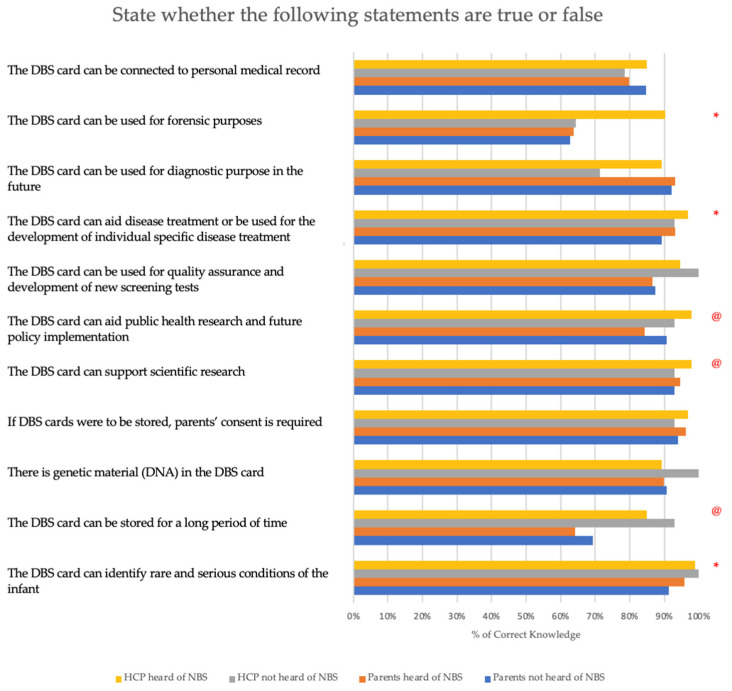
Knowledge about potential benefits of storing dried blood spot cards. * denotes statistically significant differences according to awareness of NBS; @ denotes statistically significant differences according to education level.

Figure 1 describes the knowledge about potential benefits of storing a DBS card. Overall, healthcare professionals demonstrated a higher understanding when compared to parents, with a mean of 10.2 and 9.43 correct answers out of 11 items, respectively (*p* < 0.001). While most parents (95.1%) acknowledged that their consent is required to keep their child’s DBS card, and that the DBS card supports scientific research (93.8%), identifies rare and serious conditions in babies (93.6%), provides diagnostic purposes (92.7%), aids disease treatment (91.4%), contains genetic material (90.3%) and can be connected to personal medical record (82.1%), fewer parents knew that the DBS card could be stored for a long time (66.6%) or be used for forensic purposes (63.3%). Among HCPs, 99.1% knew that DBS cards could be used to identify rare and serious conditions in babies, and 96.3% acknowledged that their consent is required to keep their child’s DBS card. They were aware that DBS card could be used in scientific research (97.2%), quality assurance (95.3%), aid disease treatment (96.3%), and contains genetic material (90.7%). Around one-sixth did not know that the DBS can be connected to personal medical records (15.9%), stored for a long time (14.0%), and be used for forensic purposes (13.1%) or diagnostic purposes (13.1%)

Subgroup analysis showed that lack of prior exposure to NBS for uncommon disorders and lower educational background contribute to the knowledge gaps. Compared with those who had heard of NBS for uncommon diseases, those who had not heard about the test were not aware of the use of DBS for identifying rare and severe conditions (*p* = 0.009), forensic purposes (*p* = 0.024), supporting the development of individual-specific disease treatment (*p* = 0.03). Respondents who attained an education level below secondary schools did not know the DBS card could be stored for an extended period (*p* < 0.001), support scientific research (*p* = 0.033), and aid public health research and future policy implementation (*p* = 0.030). No significant difference was observed by any other demographics.

### 3.4. Concerns and Views about Storing Dried Blood Spot Cards

**Figure 2:**
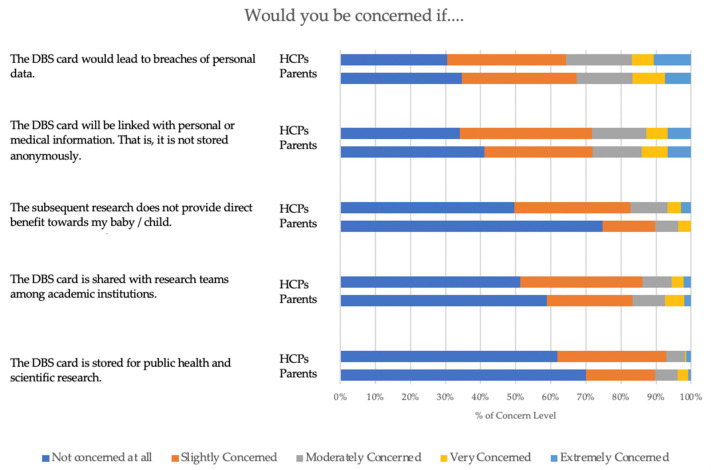
Concerns about storing dried blood spot cards. HCPs = healthcare providers.

Regarding concerns about DBS card storage (see Figure 2), about half of the parents (51.3%) and HCPs (58.9%) were not concerned about sharing DBS among academic institutions. Over 70% of parents and 60% of HCPs demonstrated considerable concerns about potential privacy breaches and anonymity. Compared to their lower-educated counterparts, the more educated expressed statistically significant concerns about the DBS being linked with personal or medical records (*p* = 0.005) or privacy breaches (*p* < 0.001). While over 70% of HCPs expressed no concern with regard to the DBS being used for secondary research, more parents (50.5%) than HCPs (25.2%) were of the opinion that the DBS secondary research does not benefit their child and are thus more reluctant to store the card for public health or scientific research purposes (*p* < 0.005). Pearson correlations reveal a negative relationship between knowledge of keeping DBS and concerns about altruistic benefits (correlation coefficient = −0.125, *p* = 0.003).

Table 2 shows views about storing DBS cards. Most parents and HCPs (72.3%) showed interest in storing DBS cards for longer than six months, which is the current protocol in public hospitals. Of these, 21.1% support storage for up to 2 years, 15.9% support it for up to 5 years, 17.9% support it for up to 18 years, and 17.4% support storing the DBS card infinitely. The only significant difference is observed by education groups: respondents who obtained post-secondary education or higher were more supportive of longer storage of more than six months (77.7%) than their lower-educated counterparts (61.3%). Overall, on a scale of 1 (do not support) to 10 (strongly support), parents (mean = 8.13) and HCPS (mean = 8.26) supported the public policy in DBS card storage. No significant difference was observed by other demographics.

### 3.5. Interest in Opting for Extended Genetic Testing

Table 3 shows the assessment of interest in opting for extended genetic testing. Around 31% of parents and 43.9% of HCP have heard of extended genetic testing in NBS. The top two conditions in extended genetic testing that parents and HCPs are interested in learning are, respectively, a childhood-onset disease with treatment (87.4% and 90.7%) and a condition listed on the current medical recommended screening panel (85.6% and 87.9%). While around 77.9% of parents are interested in learning about a variant with unknown clinical implications, only three-fifths (57%) of HCPs agreed with the early disclosure of the variant (Chi = 19.5, *p* < 0.001).

When asked about the major factor driving their interest in extended genetic testing from a scale of 1 (not important at all) to 5 (very important), parents identified the potential usefulness of genetic extended testing to help shorten the diagnosis time (mean = 3.57), identify new disease genes and diagnose individuals with rare disorders (mean = 3.54) as important factors. In contrast, HCPs, put greater emphasis on the accuracy of test results (mean = 4.32) and access to existing treatment (mean = 4.31).

Overall, on a scale of 0 (do not support) to 10 (strongly support), parents (mean = 7.87) and HCPS (mean = 7.64) supported extended genetic testing. No significant difference found between parents who have a child with a disease and other demographics.

## 4. Discussion

DBS unquestionably provides us with an easy way to store a wealth of data that can be used for many secondary purposes and extended genetic testing. The sensitive information it holds has resulted in many debates amongst experts worldwide regarding the duration of storage, secondary usage, and research policies and how and when to consent to DBS storage and its secondary uses. This has resulted in variable global policies. There, however, is a trend to store DBS cards for a prolonged period of time and to use them for biobanking. In New Zealand [25], some states in the US, such as Minnesota [26,27], and Victoria in Australia [28], DBS cards are stored indefinitely. In Denmark, the residual DBS cards are stored at the Danish Newborn Screening Biobank indefinitely [29]. The United Kingdom stores DBS cards for 5 years [30].

The opt-in NBS for inherited metabolic diseases and severe combined immunodeficiency for newborns born in public hospitals is funded by the Hong Kong Government. DBS samples were collected for NBS purposes and archived for six months after reporting except for quality assurance purposes. The issues about DBS storage and its secondary use are yet to be explored. Ethical questions and consenting logistics remain challenges. However, before moving on, it is essential to know the receptivity of our key stakeholders, i.e., the parents and HCPs in Hong Kong, toward the retention of DBS cards and their secondary usage, including extended genetic testing, and this was the purpose of our study.

Our study has shown that the awareness of NBS locally is still low, with only 52.4% of parents and 86.9% of HCPs being aware of NBS. In the Netherlands, 98.5% of mothers were aware of NBS [11], and in Australia, 93% of mothers had heard of NBS [31]. Having said that, our study includes parents who have (58.7%) heard of NBS had taken part in in NBS, and this may account for the difference. Our figures are more comparable to a Japanese study examining mothers and an American study examining the general public regarding their awareness of NBS, and these studies showed that only 57% and 55%, respectively, were aware of NBS [13,32]. Another reason for the low awareness is that NBS has only been established territory wide in Hong Kong in the last five years, and it will take the public more time to become more aware of NBS.

This lack of awareness is also reflected in the lack of knowledge about DBS storage. Only 66.6% of parents and 86% of HCPs were aware that DBS could be stored for a long time. Despite not knowing about the prolonged storage of DBS, parents were generally aware that DBS contains genetic material (90.3%), could be used for quality assurance (86.9%) and scientific and public health research (93.8%), and was connected to personal medical record (82.1%), but few (63.3%) knew that it could be used to for forensic purposes. Lack of knowledge and awareness may be the reason that Hui et al. [16] noted the lack of support of local parents in using DBS to trace victims (69%) or suspects of crimes (52%), while they were more supportive of using DBS for quality control (85%) and health-related research (75%). This is in contrast to the Dutch population, where NBS has been established since 1974, and the population is highly supportive of using DBS to help trace suspects of serious crimes, with a rating mean of 3.63 on a 5-point scale [11]. In contrast to parents, our HCPs (86.9%) were more aware of DBS’s secondary uses and forensics uses of DBS (86.9%). Thus, our frontline HCPs should be recruited as collaborative partners to help fill the knowledge gap of the parents since our respondents (61.2%) considered HCPs to be the most credible source of information.

The concerns of both HCPs (65.4%) and parents (69.5%) relating to the storage of DBS with regard to breaches of personal data are very reasonable concerns to have, considering the sensitivity of information the DBS contains about an individual. This concern only reiterates that strict data protection policies need to be put into place to gain the trust of our parents and HCPs if prolonged DBS storage is to be considered in Hong Kong.

Interestingly, the parents (50.5%) in Hong Kong did not seem to be as altruistic as our HCP (25.2%) when it came to research, and they were concerned that the research did not directly benefit their child/baby. Bombard et al. [1] showed that among Canadian citizens, 77% supported anonymous research. Further analysis revealed that Canadian citizens’ acceptance of or discomfort with a routinised approach to research with stored samples varied along axes of trust, concern for harm, and individual versus collective interests. There is a need for a more focus group-based research locally to understand parent’s motivations for supporting and the reservations to use DBS for secondary research.

An important issue that our study did not discuss extensively is consent. Storage policies are an opt-out option in New Zealand, Minnesota (US), Denmark, and the United Kingdom. In New Zealand, Denmark, and Minnesota (US), the secondary use of DBS, including research, is mentioned on the leaflet for NBS or the website for NBS. In the United Kingdom, no consent for de-identifiable blood spots is necessary. Victoria in Australia uses an opt-in approach, and written consent for the de-identified secondary use and health research is taken during the NBS consent. Denmark has a clear policy and states the purpose of storage for the (1) diagnosis and treatment of congenital disorders, including documentation, repeat testing, quality assurance, statistics, and improvement of screening methods; (2) diagnostic use later in infancy after informed consent; (3) legal use after a court order; and (4) the possibility of research projects after approval by the Scientific Ethical Committee System in Denmark, The Danish Data Protection Agency and the NBS-Biobank Steering Committee. Hui et al. showed that Hong Kong parents prefer the opt-in approach rather than the opt-out approach [16]. More research is required to understand local preferences.

Regarding the length of storage of DBS, keeping their concerns aside, 17.5% of parents and 19.6% of HCPs were willing to store the DBS up to 18 years of age, and 19.2% of parents and 9.3% of HCPs were supportive of storing the DBS indefinitely. Overall, 72.3% of parents and HCPs supported storing DBS beyond the current 6-month practice, and this is a promising start for any bio-banking future in Hong Kong.

In the US, various projects are underway to test the usefulness of whole-exome sequencing (WES) or whole-genome sequencing (WGS) as a tool in newborn sequencing—Babyseq project [33], NC Nexus [34], etc. The United Kingdom National Health Service is also exploring the benefits of next-generation sequencing in newborn screening [35]. All of these programs have discovered the benefits of extended genetic testing in NBS, including the reduction in false-positive and false-negative rates [36], the discovery of conditions not identified by MS/MS newborn screening [34,37], and even shortening time to diagnosis [38]. Extended genetic testing in NBS is the future that will radically change the face of health screening worldwide. Goldenberg et al. showed that 74% of parents were interested in WGS in newborns [39]. This is similar to our results in which 78.1% of parents and 73.4% of HCPs supported extended genetic testing in NBS.

However, apart from identifying medically actionable childhood-onset disease, it can also identify actionable adult-onset disease. This may have a huge psychological impact on the individual and their family members regarding the uncertainty of the time of onset of the disease. Extended genetic testing can also detect non-actionable conditions and conditions of unknown significance. Our parents (90.7%) and HCPs (87.4%) were more interested in knowing about actionable conditions and less about non-actionable conditions. This is different from the Western population, where Lewis et al. showed that with regard to non-actionable conditions, parents preferred to learn about disorders with more severe manifestations, even though this may result in increased distress [40]. Our parents and HCPs were well aware that a genetic diagnosis alone is inadequate, and thus, the factors which will influence their interest in extended genetic testing include the accuracy of test results, access to specialist follow-up and waiting time length, and access to existing treatment without any impingement on an open future. These are all important factors for a society to consider before embarking on including extended genetic screening in their NBS program. More small group analysis is required to comprehend better the societal and ethical preferences of local HCPS and parents.

## 5. Conclusions

In conclusion, the establishment of DBS-based NBS in Hong Kong started in 2015. The issues of DBS storage and secondary usages, including extended genetic testing, need to be further explored, hoping to be in line with other countries. Our study shows that addressing the clinical, ethical, and social concerns of HCPs and parents is paramount to making the leap forward. Thus, more research is needed to understand the local preferences and views. In the meantime, we will continue to engage our HCPs to enhance the education of parents about NBS and DBS storage and its secondary uses in order to fill the knowledge gap and conduct more research to understand local preferences.

## 6. Strengths and Limitations

There are several limitations to the current study. First, this was a cross-sectional assessment of attitudes toward the retention of DBS cards and their usage for extended genetic testing. We compared groups of parents and HCPs, but the results should be interpreted carefully at the community level as sociodemographic and professional characteristics observed individual variances. Second, the study did not report the baseline knowledge of medical genetics, which may have affected respondents’ views of extended genetic testing. Third, the sample consisted of those who responded to our survey and are willing to participate in a study in the public sector only, and we have no private sector data for comparison. Finally, the survey instrument’s closed-ended questionnaire structure did not allow additional ethical concerns to be raised. Thus, the data do not fully describe the ethical considerations in the sample population but rather their views about predetermined issues.

## Figures and Tables

**Table 1:** Demographics of survey respondents.

	All	Healthcare Providers	Parents	Chi/t	*p*-Value
	(*n* = 559)	(*n* = 107)	(*n* = 452)		
	*n* (%)	*n* (%)	*n* (%)		
**Age**					
mean (SD)	36.8 (6.57)	39.39 (10.33)	36.17 (5.18)	3.06	<0.001
**Gender**				ns	ns
Female	412 (73.7%)	76 (71%)	336 (74.3%)		
Male	147 (26.3%)	31 (29%)	116 (25.7%)		
**Ethnicity**				15.6	<0.001
Hong Kong Chinese	500 (89.5%)	107 (100%)	393 (86.9%)		
Mainland Chinese	32 (5.7%)	-	32 (7.1%)		
Other	27 (4.8%)	-	27 (6%)		
**Religion**				31.25	<0.001
None	370 (66.2%)	52 (48.6%)	318 (70.4%)		
Christian	111 (19.8%)	39 (36.4%)	72 (15.9%)		
Catholic	39 (7.1%)	12 (11.2%)	27 (6%)		
Buddhist	26 (4.6%)	4 (3.8%)	22 (4.8%)		
Other	13 (2.3%)	-	13 (2.9%)		
**Do you work in a health-care related field?**					
No	-	-	409 (90.5%)	-	-
Yes	-	-	43 (9.5%)	-	-
**Profession**					
Doctor	-	34 (31.8%)	-	-	-
Nurse	-	36 (33.6%)	-	-	-
Laboratory Technician	-	5 (4.7%)	-	-	-
Others	-	32 (29.9%)	-	-	-
**Years of Experience in NBS**				-	-
Mean (SD)	-	5.49 (7.35)	-		
**Highest Education**				118.7	<0.001
Upper Secondary or lower	174 (31.1%)	2 (1.9%)	172 (38.0%)		
Post-Secondary	78 (14.0%)	3 (2.8%)	75 (16.6%)		
Tertiary	197 (35.2%)	47 (43.9%)	150 (33.2%)		
Master or above	110 (19.7%)	55 (51.4%)	55 (12.2%)		
**Family Income**				89.55	<0.001
Below HKD 19,999	93 (16.7%)	3 (2.8%)	90 (19.9%)		
HKD 20,000–29,999	81 (14.5%)	1 (0.9%)	80 (17.7%)		
HKD 30,000–39,999	89 (15.9%)	6 (5.6%)	83 (18.4%)		
HKD 40,000–49,999	37 (6.6%)	4 (3.7%)	33 (7.3%)		
Above HKD 50,000	259 (46.3%)	93 (87.0%)	166 (36.7%)		
**Consanguinity**					
No	-	-	424 (93.8%)	-	-
Yes	-	-	24 (5.3%)	-	-
Not Sure	-	-	4 (0.9%)	-	-
**Kid Number**					
1	-	-	198 (43.8%)	-	-
2	-	-	202 (44.7%)	-	-
3 or above	-	-	52 (11.5%)	-	-
**Diagnosed Disease**					
None	-	-	291 (64.4%)	-	-
Cardiac Diseases	-	-	40 (8.8%)	-	-
Haematology	-	-	21 (4.7%)	-	-
Respiratory	-	-	16 (3.5%)	-	-
Neonatology	-	-	15 (3.3%)	-	-
Endocrine Diseases	-	-	14 (3.1%)	-	-
Neurology	-	-	9 (2.0%)	-	-
Metabolic Diseases	-	-	8 (1.8%)	-	-
Others	-	-	38 (8.4%)	-	-

ns = not significant.

**Table 2:** Views toward storing dried blood spots.

	All	Healthcare Providers	Parents	Chi/t	*p*-Value
	(*n* = 559)	(*n* = 107)	(*n* = 452)		
	*n* (%)	*n* (%)	*n* (%)		
**How long do you think DBS should be stored?**					
Up to 6 months	155 (27.7%)	29 (27.2%)	126 (27.9%)	ns	ns
Up to 2 years	118 (21.1%)	23 (21.5%)	95 (21%)	ns	ns
Up to 5 years	89 (15.9%)	24 (22.4%)	65 (14.4%)	ns	ns
Up to 18 years	100 (17.9%)	21 (19.6%)	79 (17.5%)	ns	ns
Indefinitely	97 (17.4%)	10 (9.3%)	87 (19.2%)	ns	ns
**Do you support DBS storage? (1 as not interested at all; 10 as extremely interested)**					
mean (SD)	8.15 (1.66)	8.26 (1.70)	8.13 (1.65)	ns	ns

ns = not significant.

**Table 3:** Interest in opting for extended genetic testing.

	All	Healthcare Providers	Parents	Chi	*p*-Value
	(*n* = 559)	(*n* = 107)	(*n* = 452)		
	*n* (%)	*n* (%)	*n* (%)		
**If you were the parent, would you wish to receive the following information with regard to your child/If you were a healthcare provider, would you wish to disclose the following information to parents?**					
A childhood-onset disorder is a condition with treatment	492 (88.0%)	97 (90.7%)	395 (87.4%)	ns	ns
A condition list on the current medical recommended screening panel	481 (86.0%)	94 (87.9%)	387 (85.6%)	ns	ns
Increase risk for an adult-onset disease	467 (83.5%)	83 (77.6%)	384 (85.0%)	ns	ns
The newborn is a carrier for the condition but will not develop it	470 (84.1%)	93 (86.9%)	377 (83.4%)	ns	ns
An adulthood-onset disorder is a condition with treatment	466 (83.4%)	94 (87.9%)	372 (82.3%)	ns	ns
A childhood-onset disorder is a condition with NO available treatment	456 (81.6%)	91 (85.0%)	365 (80.8%)	ns	ns
A variant that has unknown clinical implications	413 (73.9%)	61 (57.0%)	352 (77.9%)	19.5	<0.001
An adulthood-onset disorder is a condition with NO available treatment	421 (75.3%)	81 (75.7%)	340 (75.2%)	ns	ns
**What is the factor(s) impacting your interest in extended genetic testing? (1 is not important at all; 5 is extremely important)**					
	mean (SD)	mean (SD)	mean (SD)	t	*p*-value
Shorten the diagnosis time in future	3.69 (1.17)	4.20 (0.884)	3.57 (1.19)	6.11	<0.001
Identify new disease genes and diagnose individuals with rare disorders	3.66 (1.16)	4.14 (0.976)	3.54 (1.17)	5.45	<0.001
Access to existing treatment for affected individuals	3.68 (1.15)	4.31 (0.862)	3.53 (1.16)	7.84	<0.001
Accuracy of the test results/sequencing results	3.67 (1.19)	4.32 (0.938)	3.52 (1.20)	7.52	<0.001
Access to specialist follow-up for affected individuals	3.64 (1.15)	4.18 (0.92)	3.51 (1.17)	6.38	<0.001
Impinge on the child’s right to an open future	3.58 (1.19)	4.06 (0.96)	3.47 (1.21)	5.37	<0.001
Diagnosing susceptibility to adult-onset disease during the newborn period	3.35 (1.18)	3.45 (1.27)	3.32 (1.16)	ns	ns
**How interested would you be in obtaining your child’s extended genetic testing? (1 as not interested at all; 10 as extremely interested)**					
mean (SD)	7.83 (1.85)	7.64 (1.87)	7.87 (1.85)	ns	ns

ns = not significant.

## Data Availability

The data supporting this study’s findings are available on reasonable request from the corresponding author.

## Supplementary Materials

The survey questionnaires can be downloaded at: https://www.mdpi.com/article/10.3390/ijns9030045/s1.
