# Supplementary material for: Public and Healthcare Provider Receptivity toward the Retention of Dried Blood Spot Cards and Their Usage for Extended Genetic Testing in Hong Kong
Source: Int J Neonatal Screen. 2023 Aug 11;9(3):45. doi: 10.3390/ijns9030045 (PMC10443280; doi:10.3390/ijns9030045)
Supplement: Supplementary file 1 [file IJNS-09-00045-s001.zip › Supplementary Materials_Survey (Healthcare Providers).pdf]

Views towards dried blood spot cards storage and its usage for extended genetic testing among parents and healthcare providers in Hong Kong

(Healthcare Providers)

The survey aims to gather attitude towards dried blood spot card storage and extended genetic testing among parents, informing public policy development in future. The study is funded by the Health and Medical Research Fund, the Food and Health Bureau and conducted by the Hong Kong Children's Hospital, in collaboration with Pamela Youde Nethersole Eastern Hospital, Princess Margaret Hospital, and the Faculty of Medicine of the University of Hong Kong.

The survey is anonymous, and your participation is entirely voluntary. All information that you provide is kept secure. It approximately takes about 10-15 minutes. The study has been reviewed by the Hong Kong Children's Hospital Research Ethics Committee, Kowloon West Cluster Research Ethics Committee, and Hong Kong East Cluster Research Ethics Committee.

Part IA Information about you

1. Your age: __________ years old

2. Gender: ☐ Male ☐ Female

3. What is your ethnicity?
☐ Chinese (Hong Kong) ☐ Chinese (Mainland China) ☐ Other, please specify ________________

4. Are you a Hong Kong permanent resident?
☐ Yes ☐ No, How long have you been living in Hong Kong? ___________ years

5. What is your religious affiliation?
☐ None, I have no religious preference
☐ Christian
☐ Catholic
☐ Buddhist
☐ Other, please specify ____________

6. What is your highest level of education achieved?
☐ Primary school ☐ Lower secondary school (Secondary 1-3)
☐ Upper secondary school (Secondary 4-7) ☐ Post-secondary (diploma/ associate degree)
☐ Bachelor degree ☐ Master's degree or above

7. What is your family household monthly income (HKD)?
☐ Below $10,000 ☐ $10,000-$19,999 ☐ $20,000-$29,999
☐ $30,000-$39,999 ☐ $40,000-$49,999 ☐ $50,000 or above

Part IB Information about your training and profession

1. What is your profession? (Tick all that apply)
☐ Doctor ☐ Nurse ☐ Other, please specify: ____________
☐ Laboratory Technician ☐ Scientific Officer

2. Please specify your speciality(ies). (Tick all that apply)
☐ General Paediatrics ☐ Metabolic Medicine ☐ Neonatology
☐ Paediatric Surgery ☐ Chemical Pathology ☐ Other, please specify: ____________

3. Where are you currently practising?
☐ Public Hospital ☐ Other, please specify: __________________________

4. Are you involved in the Newborn Screening?
☐ No → Please go to Part II
☐ Yes → Please go to Q. 4.1

4.1 Which of the following parts have you involved in? (Tick all that apply)

- Provide Consultation
- Conduct laboratory test and analysis
- Provide educational sessions
- Inform parents result (Call-back)
- Other, please specify: ____________

4.2 How long have you been in newborn screening practice? __________ Years

Part IIA Understanding of Newborn Screening and Dried Blood Spots Card Storage

Description of Newborn Screening with Dried Blood Spots Card (DBS card)

Shortly after the baby infant is born, a health professional will perform a heel prick to collect a few drops of blood on a special card. The DBS card will be sent to the laboratory for the screening of some rare but serious conditions which could be picked up and treated early with benefit the affected babies in the long run. The DBS card also increasingly secondary uses, such as diagnosis of infective conditions, patient specific treatment, scientific research and extended genetic testing.

DBS Cards are stored by various countries for various duration ranging from 6 months to indefinitely after newborn screening has been completed for secondary uses i.e. quality assurance.

1. Have you heard about Newborn Screening for uncommon disorders in Hong Kong?

- No → Please go to Q. 2
- Yes → Please go to Q. 1.1

1.1 From which of the following sources have you heard of Newborn Screening? (Check all that apply)

- Hospital websites or brochure
- Healthcare provider, including doctors and nurses
- Research papers
- Other parents
- Media (i.e., newspaper, magazine)
- Websites, online platforms
- Others, please specify: ____

1.2 From your experience, at which time point should the information about the Newborn Screening given to parents?

- First Trimester Pregnancy (1-12 weeks)
- Second Trimester Pregnancy (13-26 weeks)
- Third Trimester Pregnancy (27-40 weeks)
- Soon after the birth delivery (First or second day)
- Not Sure

1.3 Did you think Newborn Screening was useful?

- No
- Yes

2. This section asks about your understanding of the DBS card. Please decide whether the following sentences are true or false to the best of your knowledge. (Please tick appropriate box)

a)	The DBS card can identify rare and serious conditions of the infant	True	False
b)	The DBS card can be stored for a long period of time	True	False
c)	There is genetic material (DNA) in the DBS card	True	False
d)	If DBS cards were to be stored, parents' consent is required	True	False
e)	The DBS card can support scientific research	True	False
f)	The DBS card can aid public health research and future policy implementation	True	False
g)	The DBS card can be used for quality assurance and development of new screening tests	True	False
h)	The DBS card can aid disease treatment or be used for the development of individual specific disease treatment	True	False
i)	The DBS card can be used for diagnostic purpose in the future (i.e. neonatal infection with presentation in infancy / childhood)	True	False
j)	The DBS card can be used for forensic purposes	True	False
k)	The DBS card can be connected to personal medical record	True	False

3. **The following lists out concerns related to the storage of DBS card.** Please use the following scale to indicate how much you concern with each statement below from 1= Not at all to 5= Extremely concerned. (Please tick appropriate circle)

Statements	Not Concerned At All	-----	-----	-----	Extremely concerned
a) The DBS card is stored for public health and scientific research. Would you be concerned?	☐ 1	☐ 2	☐ 3	☐ 4	☐ 5
b) The DBS card is shared with research teams among academic institutions. Would you be concerned?	☐ 1	☐ 2	☐ 3	☐ 4	☐ 5
c) The DBS card will be linked with personal or medical information. That is, it is not stored anonymously. Would you be concerned?	☐ 1	☐ 2	☐ 3	☐ 4	☐ 5
d) The subsequent research does not provide direct benefit towards the infant / child. Would you be concerned?	☐ 1	☐ 2	☐ 3	☐ 4	☐ 5
e) Would you be concerned if the DBS card would lead to breaches of personal data?	☐ 1	☐ 2	☐ 3	☐ 4	☐ 5

4. How long do you think residual DBS card should be stored?
   - ☐ Up to 6 months (e.g. current HA policy)
   - ☐ Up to 2 years (e.g. Texas, U.S.)
   - ☐ Up to 5 years (e.g. Netherland)
   - ☐ Up to 18 years (e.g. NSW, Australia)
   - ☐ Indefinitely (e.g. Denmark)

5. Overall, would you support the DBS card storage? (Please circle the score that best fits your feelings)

0	1	2	3	4	5	6	7	8	9	10

Not support at all **Very supportive**

Part IIB Understanding of Extended Genetic Testing

Description of Extended Genetic Testing

It is possible to study a person's entire genome extracted from DBS card. This process is called extended genetic sequencing. It may give you information about a person's risk of having different diseases both in the present and in the future, including childhood diseases such as epilepsy and adult onset diseases breast cancer or diabetes.

Imagine that you have a recently born baby. Newborn Screening program now offers you the chance to get extended genetic sequencing done for your baby. You would receive the results and would not have to pay for the testing. You can decide whether or not you want the information to be a part of your child's medical record.

1. Have you heard of extended genetic testing in Newborn Screening?
   ☐ No ☐ Yes

2. Below are some scenarios describing the return of extended genetic testing results. Would you wish to disclose the following information to parents? (Please tick appropriate box)

a)	The newborn has a condition listed on the current medical recommended uniform screening panel (USA)	☐ No	☐ Yes
b)	The newborn is a carrier for a condition. Carriers do not develop the condition but pass the mutation to future generations	☐ No	☐ Yes
c)	The newborn has a childhood-onset disorder that has treatment implications (medically actionable) i.e epilepsy	☐ No	☐ Yes
d)	The newborn has a childhood onset disorder with NO available treatment (NOT medically actionable) i.e. Lowe syndrome	☐ No	☐ Yes
e)	The newborn has an adult onset disorder that has treatment implications (medically actionable) i.e hereditary breast and ovarian cancer	☐ No	☐ Yes

f)	The newborn has an adult onset disorder with NO available treatment (NOT medically actionable) i.e. Alzheimer's disease and Huntington's disease	☐ No ☐ Yes
g)	The newborn has mutations which increase the risk for an adult-onset disease i.e. heart disease, diabetes	☐ No ☐ Yes
h)	The newborn has mutations with unknown clinical implications	☐ No ☐ Yes

3. What is the factor(s) influencing your interest in offering extended genetic testing? Please use the following scale to indicate how important each factor would be in your decision to have testing from 1 = Not at all to 5 = Very important. (Please circle appropriate number)

4.

	**Factors**	**Not Important At All**				**Very Important**
a)	Accuracy of the test results/ sequencing results	☐ 1	☐ 2	☐ 3	☐ 4	☐ 5
b)	Diagnosing susceptibility to adult onset disease (i.e. breast cancer / colon cancer / diabetes) during newborn period	☐ 1	☐ 2	☐ 3	☐ 4	☐ 5
c)	Access to specialist follow-up for affected individuals	☐ 1	☐ 2	☐ 3	☐ 4	☐ 5
d)	Access to existing treatment for affected individuals	☐ 1	☐ 2	☐ 3	☐ 4	☐ 5
e)	Shorten the diagnosis time in future	☐ 1	☐ 2	☐ 3	☐ 4	☐ 5
f)	Identify new disease genes and diagnose individuals with rare disorders	☐ 1	☐ 2	☐ 3	☐ 4	☐ 5
g)	Impinge on the child's right to an open future	☐ 1	☐ 2	☐ 3	☐ 4	☐ 5

5. Would you be interested in offering extended genetic testing for parents?

0	1	2	3	4	5	6	7	8	9	10

Thank you for your time and interest in completing this questionnaire!
